# Combination Therapy with Gossypol Reveals Synergism against Gemcitabine Resistance in Cancer Cells with High BCL-2 Expression

**DOI:** 10.1371/journal.pone.0050786

**Published:** 2012-12-04

**Authors:** Foong Ying Wong, Natalia Liem, Chen Xie, Fui Leng Yan, Wing Cheong Wong, Lingzhi Wang, Wei-Peng Yong

**Affiliations:** 1 Department of Haematology-Oncology, National University Health System, Singapore, Singapore; 2 Bioinformatics Institute, Agency for Science, Technology and Research, Singapore, Singapore; 3 Cancer Science Institute of Singapore, National University of Singapore, Singapore, Singapore; Bauer Research Foundation, United States of America

## Abstract

Although gemcitabine is highly active in several cancer types, intrinsic and acquired drug resistance remains a major challenge. Overexpression of Bcl-2 has been associated with gemcitabine resistance. The aim of this study is to determine whether gossypol can overcome gemcitabine resistance in cell lines with high level of Bcl-2 expression in combination drug therapy. Our study demonstrated that in 10 cell lines derived from different cancers, high Bcl-2 baseline expression was observed in cell lines that were resistant to gemcitabine (GEM-R). Furthermore, synergistic effect of combination therapy was observed in gemcitabine-resistant (GEM-R) cell lines with high Bcl-2 expression, but not in a gemcitabine-sensitive (GEM-S) cell lines regardless of Bcl-2 expression. Gossypol treatment resulted in the decrease of anti-apoptotic genes such as Bcl-2 and Bcl-xl and an upregulation of the pro-apoptotic gene, Noxa. Furthermore, the addition of gossypol to gemcitabine resulted in lower expressions of anti-apoptotic genes compared to gemcitabine alone. Gene expression profiling in GEM-R and GEM-S cell lines suggest that anti-apoptotic genes such as pAkt and PI3KR2 may play important role in gemcitabine resistance, while pro-apoptotic Bcl-2 related genes (Bad, Caspase-6 and Calpain-1) may regulate synergistic interaction in combination therapy.

## Introduction

Gemcitabine (2′, 2′-difluorodeoxycytidine, dFdC) is a pyrimidine nucleoside analogue of deoxycytidine commonly used in breast [1], non-small cell lung [2], pancreatic [3] and ovarian cancer [4]. Gemcitabine enters the cell via specific nucleoside transporter and undergoes intracellular phospharylation to convert into active drug metabolites. Once inside the cell, gemcitabine is phosphorylated by deoxycytidine kinases which then incorporate into DNA to inhibit synthesis and cell proliferation, whilst promoting apoptosis in cancer cells [5]. Overexpression of anti-apoptotic genes, such as the Bcl-2 family play a critical role in conferring resistance to conventional anticancer therapies [6], [7], [8]. For instance, *Yu et al* demonstrated an inverse correlation between Bcl-2 expression and chemosensitivity to several drugs including adriamycin and 5-Fluorouracil in breast cancer cells. [9]. *In vitro* cultured cell lines derived from primary HCC tumours that express high levels of the anti-apoptotic Bcl-2 and Bcl-xl were also found to be resistant to paclitaxel [10]. Up-regulation of Bcl-2 family has been implicated in intrinsic gemcitabine resistance in pancreatic and lung cancers [11], [12]. Pancreatic cancer cells that acquired drug resistance to gemcitabine after continuously exposed to gemcitabine had up-regulation of the anti-apoptotic genes such as Bcl-xl and Mcl-1 [13]. These findings suggest that elevated level of Bcl-2 family may play an important role in the development of gemcitabine resistance during chemotherapy.

Gossypol is a polyphenolic compound isolated from the cotton plant (*Gossypium Malvaceae*). Initially used as a male fertility-control agent, it was recently discovered to have anti-tumorgenic properties in a variety of cancers [14]. Gossypol exists in two enantiomeric forms, (+) and (−), and naturally occurring gossypol exists as a racemic mixture of (+) and (–) enantiomers. The (–) enantiomer of gossypol has been found to possess more potent cytotoxic effect than (+) enantiomer or racemic gossypol. Gossypol is a BH3 mimetic that inhibits the function of anti-apoptotic Bcl-2 proteins through interactions with the BH3-binding pockets of Bcl-2 and Bcl-xl proteins [15]. Several in vitro studies have reported that gossypol could inhibit cell growth in a wide spectrum of cancers including colon [16], prostate [17], lung [18] and breast tumours [19]. In PC-3 prostate cancer cells, gossypol induced apoptosis by inhibiting the heterodimerization of Bcl-2/Bcl-xl complex [17]. Induction of apoptosis via gossypol was also observed by the down-regulation of anti-apoptotic Bcl-2 family proteins in HT-29 colon cancer and chronic lymphocytic leukemia [16], [20]. In addition, gossypol has the ability to overcome drug resistance to other conventional chemotherapeutic drugs. Gossypol has been shown to effectively induce cell death in chemoresistant leukemia cells lines overexpressing Bcl-2 and Bcl-xl [21]. It had been reported that gossypol could induces apoptosis with high efficiency in cisplatin-resistant head and neck cancer cell lines that express high levels of Bcl-xl. [22]. Cengiz E et al. observed that the combined treatment of gossypol and docetaxel could synergistically induced apoptosis in PC-3 prostate cancer cell line [23]. These studies suggested that the combination of gossypol with other drugs may improve the efficiency of inducing apoptosis in cancer therapy.

The aims of this study were to (1) correlate Bcl-2 protein expression with gemcitabine resistance (GEM-R) in gastric, nasopharyngeal and breast cancer cell lines, (2) evaluate the combination of gemcitabine and gossypol in GEM-R cancer cell lines with high level of Bcl-2 expression and (3) determine the mechanisms involved in reversing gemcitabine resistance by gossypol.

## Materials and Methods

### Cell Lines and Reagents

Nasopharyngeal cancer cell lines CNE1, CNE2, HONE1 were all derived from undifferentiated Chinese NPC tumors, while HK1 was from a well-differentiated Chinese NPC [24], [25]; breast cancer cell lines SKBR3, T47D, MCF-7 and gastric cancer cell lines AGS, SNU1 were purchased from ATCC, USA; while YCC16 was obtained from DUKE NUS, Singapore, All cell lines were cultured at 37°C in humidified atmosphere containing 5% CO_2_ and maintained in RPMI 1640 medium (Gibco; Grand Island, NY) containing 10% heat inactivated fetal bovine serum (Gibco; Grand Island, NY) and 1% penicillin/streptomycin (Gibco; Grand Island, NY). Mycoplasma status of cells was routinely tested using MycoAlert™ Mycoplasma Detection Kit (Lonza; ME, USA). Mycoplasma status of all the cell lines carried out in this study were negative. Gossypol (Sigma-Aldrich; St Louis, MO) and gemcitabine (Sigma-Aldrich; St Louis, MO) were dissolved in DMSO at a concentration of 10 mM and stored at −20°C. AT-101 was provided by Ascenta Therapeutics and prepared as 10 mM stock with DMSO. Primary antibodies consisting of anti-caspase-6, anti-Bcl-xl, anti-Bad, anti-Bak, anti-Bax, anti-pAKT, anti-PIK3R2, anti-calpain-1 and anti-Actin were purchased from Cell Signaling Technology (Cell Signaling; MA, USA). Primary anti-Noxa was purchased from Calbiochem (Merck; Germany); and anti-Bcl-2 and anti-Mcl-1 was purchased from Santa Cruz Biotechnology (Santa Cruz Biotechnology; CA, USA). Secondary antibodies (anti-rabbit IgG, HRP-Linked and anti-mouse IgG, HRP-Linked) were purchased from Cell Signaling Technology (Cell Signaling; MA, USA).

### Cell Viability and Proliferation Assays

To assess the chemosensitivity of tumor cells, cell viability was measured by MTS (Colorimetric CellTiter 96 AQ_ueous_ One Solution Cell Proliferation Assay) (Promega; WI, USA). Cell suspension was cultured in 96-well flat-bottomed microtiter plates at a concentration of 2×10^3^ cells/per well and incubated overnight. Drug treatments were carried out as follows: gossypol (0.1 µM –100 µM), gemcitabine (0.001 µM–100 µM) or a combination of both drugs in varies concentrations. Each drug was tested in triplicate. The plates were incubated at 37°C in a humidified atmosphere containing 5% CO_2_ for 72 h. Microtiter wells containing tumour cells with no drug treatments were used as controls, and wells containing only complete medium were used as blank controls for nonspecific dye reduction. Cells were incubated for 72 h before the addition of MTS solution (1 mg/mL per well) and absorbance was read at 490 nm using a spectrophotometric microplate reader (Bio-Rad; CA, USA). The percent cell viability to different drug concentrations was calculated as the inhibition rate of (mean absorbance of treated wells/mean absorbance of control wells)×100%. IC_50_ was calculated by GraphPad Prism v4.0 (GraphPad Software, Inc; CA, USA).

### Combination Index Analysis

The interaction between gemcitabine and gossypol was analyzed with Calcusyn software (Biosoft, Cambridge, UK) as previously reported [26]. Briefly, the constant concentration ratio of gemcitabine and gossypol at 0.25x, 0.5x, 1x, 2x, and 4x was used to assess the synergy of combination treatment. The CI values were calculated according to the levels of growth inhibition (fraction affected) by each agent individually and combination of gemcitabine with gossypol. Combination index was calculated to illustrate synergism (CI<0.9), antagonism (CI>1.1) and additively (CI = 0.9–1.1).

### Statistics of MTS Assay Analysis

The dosage increments were log-transformed, allowing equal horizontal spacing of data points on the dose response curve. Spline curve was plotted from fit spline/LOWESS analysis by GraphPad Prism v4.0 and the concentration resulted in 50% cell death was determined from the curve generated. IC_50_ was calculated by antilog the value obtained. All data are presented as mean ± standard error (SE) from at least two independent experiments.

### Western Blot and Protein Analysis

Cells treated with drug were washed with ice cold PBS and resuspended in lysis buffer (CelLytic; Sigma-Aldrich; St Louis, MO) in the presence of a protease inhibitor cocktail (Roche; Mannheim, Germany). Lysates were sonicated, incubated on ice for 20 min and centrifuged at 14000 rpm for 20 min at 4°C. Protein concentrations were determined using Bradford assay (Bio-Rad, CA, USA). 20 µg of protein samples were electrophoretically separated on 12% SDS-PAGE and electrotransferred to a PVDF membrane (Immun-Blot PVDF; Bio-Rad, CA, USA). Membranes were blocked for an hour at room temperature in 5% non-fat dry milk (Bio-Rad; CA, USA) and subsequently incubated with primary antibodies overnight at 4°C. The respective primary antibodies were used as follows: anti-Bcl-xl (Cell Signaling; MA, US) at 1∶1000 dilution, anti-Bad (Cell Signaling; MA, US) at 1;1000 dilution, anti-Bak (Cell Signaling; MA, US) 1∶1000 dilution, anti-Bax (Cell Signaling; MA, US) 1∶3000 dilution, anti-caspase-6 (Cell Signaling; MA, US) 1∶1000 dilution, anti-P1K3R2 (Cell Signaling; MA, US) 1∶1000 dilution, anti-pAKT (Cell Signaling; MA, US) at 1∶1000 dilution, anti-calpain-1 (Cell Signaling; MA, US) at 1∶1000 dilution, anti-Actin (loading control; Cell Signaling; MA, US) at 1∶3000 dilution, anti-Bcl-2 (Santa Cruz Biotechnology; CA, USA) 1∶3000 dilution, and anti-Mcl-1 (Santa Cruz Biotechnology; CA, USA) at 1∶3000 dilution, and anti-Noxa (Calbiochem, Merck; Germany) 1∶1000 dilution. After three washes with Tween 20 in PBS, membranes were incubated for 1 hr at room temperature with corresponding horseradish peroxidase conjugated anti-rabbit and anti-mouse secondary antibodies. The membranes were washed four times with PBS/Tween 20, and the signals were visualized by ECL reagent (AmershamTM ECL Plus Western Blotting Detection System; GE Healthcare; Buckinghamshire, UK), followed by exposure to chemiluminescence film (Amersham HyperfilmTM ECL; GE Healthcare; Buckinghamshire, UK). Immunoblot analyses were repeated at least twice for each protein tested.

### Gene Expression Arrays

Whole-genome expression profiling of 2 cancer cell lines (CNE2 and SNU1) were carried out using the HumanHT-12 v4 whole-genome gene expression direct hybridization assay (Illumina, San Diego, USA). Total RNA from cells treated with drug was extracted using the TRIzol reagent (Invitrogen, USA) as per the manufacturer’s instructions. RNA samples were quantitated using a ND-1000 spectrophotometer (Thermo Fisher Scientific, USA) and assessed for degradation using the BioAnalyzer 2100 (Agilent Technologies, USA) prior to gene expression array analysis. Total RNA (500 ng) was converted to cDNA, followed by an *in vitro* transcription to synthesis biotin-labeled cRNA using the IIumina® TotalPrep RNA Amplification Kit (Illumina, San Diego, USA) as per manufacturer’s instructions. Labeled cRNA were resuspended with hybridization reagents and hybridized to the HumanHT-12 v4 BeadChips for 18 hours in 58°C hybridization oven. The BeadChips were washed, blocked and stained to prepare for scanning. Fluorescence intensity at each probe was scanned using the Illumina BeadArray Reader. Three independent experiments were conducted for each sample.

### Statistical Selection of Post-treatment Differentially Expressed Genes

Differentially expressing genes between the drug treatment groups (gemcitabine and combination of gemcitabine and gossypol) in the two cell lines (CNE2 and SNU1 respectively) were determine based on the methodology discussed in Wong et.al [27]. For each gene, the one-way ANOVA is first applied to test for any differences between the means of the two treatment groups at a significance level of 0.05. If the null hypothesis is rejected, the Dunnett’s posthoc test was conducted to determined the differences between any two comparisons at the family-wise error rate of 0.05. The comparisons are (i) control (no treatment) versus gemcitabine treatment and (ii) control versus combination treatment. A gene is marked differentially expressed (up- or down-regulated) if at least 1 comparison is detected by the posthoc procedure. The lists of differential genes from each cell line were overlapped and mapped to KEGG pathways as previously described in DAVID database [28]. EASE value of 0.1 was set to determine the significance of gene–term enrichment in DAVID annotation system. A gene list of 14065 and 11342 differentially expressed genes from both cell lines were selected for further evaluation.

## Results

### Sensitivity of Cancer Cell Lines to Gemcitabine in Relationship with Bcl-2 Expression

**Figure 1:**
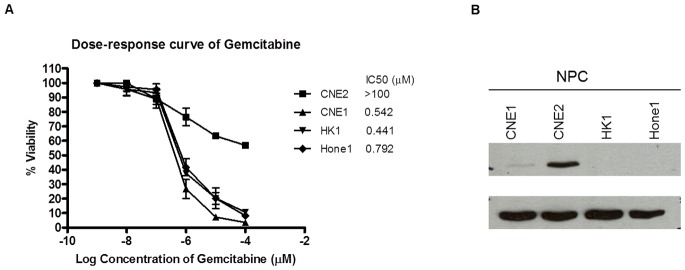
Sensitivity of nasopharyngeal cancer cell lines to gemcitabine in relationship with Bcl-2 expression. (A) IC_50_ of gemcitabine to nasopharyngeal cancer cell lines. Mean IC_50_±Standard Error (SE) of at least two independent experiments were performed in triplicates. Cells were treated with gemcitabine for 72 hr and cell proliferation was assessed using the MTS assay. (B) Immunoblot of Bcl-2 baseline expression in nasopharyngeal cancer cell lines.

**Figure 2:**
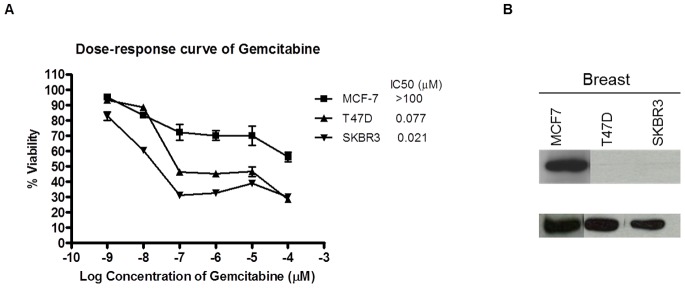
Sensitivity of breast cell lines to gemcitabine in relationship with Bcl-2 expression. (A) IC_50_ of gemcitabine to breast cancer cell lines. Mean IC_50_±Standard Error (SE) of at least two independent experiments were performed in triplicates. Cells were treated with gemcitabine for 72 hr and cell proliferation was assessed using the MTS assay. (B) Immunoblot of Bcl-2 baseline expression in breast cancer cell lines.

**Figure 3:**
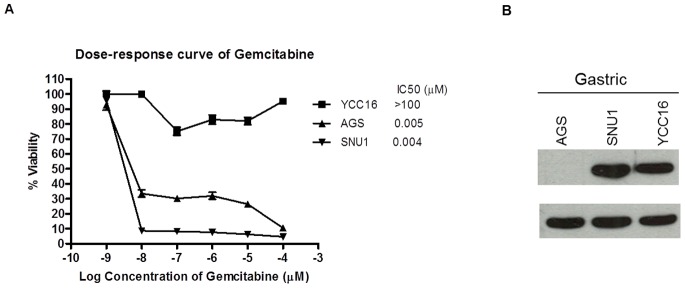
Sensitivity of gastric cell lines to gemcitabine in relationship with Bcl-2 expression. (A) IC_50_ of gemcitabine to gastric cancer cell lines. Mean IC_50_ of ±Standard Error (SE) of at least two independent experiments were performed in triplicates. Cells were treated with gemcitabine for 72 hr and cell proliferation was assessed using the MTS assay. (B) Immunoblot of Bcl-2 baseline expression in gastric cancer cell lines.

Four nasopharyngeal cancer cell lines (NPC) were treated with various concentrations of gemcitabine (0.001 to 100 µM) to evaluate the chemosensitivity of gemcitabine. There was a wide range in IC_50_ of gemcitabine sensitivity whereby CNE2 was found to be resistant to gemcitabine, with IC_50_ over 100 µM (Figure 1A) and HK1 was found to be the most sensitive to gemcitabine. To investigate whether gemcitabine resistance is related to high expression of Bcl-2, we examined the baseline expression of Bcl-2 in all NPC cell lines. It was observed that the cell line that had high Bcl-2 expression (CNE2, Figure 1B) was resistant to gemcitabine. The association between gemcitabine resistance and Bcl-2 expression was further examined in 3 breast cancer cell lines and 3 gastric cancer cell Consistently, MCF-7 breast cancer cell line and YCC16 gastric cancer cell line that had high Bcl-2 expression were resistant to gemcitabine (Figure 2 and 3). With the exception of SNU1, low level or no expression of Bcl-2 were found in cell lines that were sensitive to gemcitabine.

### Similar Drug Response to Cancer Cell Lines between Gossypol and AT101

**Figure 4:**
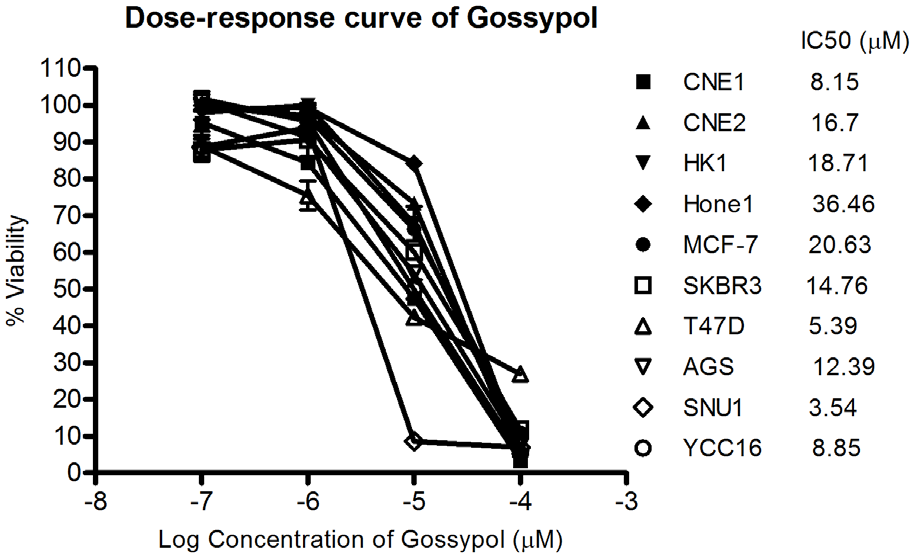
IC_50_ of gossypol in different types of cancer cell lines. Nasopharyngeal (n = 4), breast (n = 3) and gastric (n = 3) cancer cell lines were tested for their sensitivity to gossypol. Mean IC_50_ of ±Standard Error (SE) of at least two independent experiments were performed in triplicates. Cells were treated with gossypol for 72 hr and cell proliferation was assessed using the MTS assay.

Three gastric cancer cell lines (AGS, YCC16 and SNU1) were treated with gossypol and AT101 to determine their IC_50,_ respectively. As expected, (−)-gossypol was more potent than racemic gossypol but there were generally less than 10fold differences in IC_50_ between both drugs in gastric cell lines. All subsequent in vitro studies were carried out using the racemic gossypol (Table 1) as there was little difference in cellular cytotoxicity using either drug. Gossypol was found to have moderate activity in all the ten cell lines (Figure 4). Unlike gemcitabine, there was no clear association between the level of Bcl-2 expression in the cell lines to the IC_50_ of the drug.

**Table 1:** Comparison of IC_50_ between gossypol and AT101 in 3 gastric cancer cell lines.

Cell Line	IC_50_ Gossypol (µM)	IC_50_ AT-101 (µM)	p-value
YCC16	7.56±1.34	3.45±0.44	0.2096
SNU1	3.79±0.09	1.75±0.22	0.0738
AGS	12.39±0.82	7.31±0.67	0.131

Cells were treated with gossypol for 72 hr and cell proliferation was assessed using the MTS assay. Mean IC_50_ of ±Standard Error (SE) of at least two independent experiments were performed in triplicates. P-value is calculated by unpaired t test with Welch’s correction.

### Synergistic Drug Interaction between Gossypol and Gemcitabine in Gemcitabine-resistance (GEM-R) Cell Line with High Bcl-2 Expression

To determine the possible synergism interaction between gossypol and gemcitabine, combination drug treatment were carried out in all 10 cancer cell lines. With the exception of SNU1, synergism was observed in the cell lines that had high Bcl-2 expression, but not in cell lines that expressed low Bcl-2 (Table 2, Figure S1). Collectively, these observations suggest that there is a trend whereby gemcitabine resistant cell lines that expressed high level of Bcl-2 are synergistic to combination drug treatments with gemcitabine and gossypol.

**Table 2:** Synergistic drug interaction between gossypol and gemcitabine.

Cell Line	Bcl-2 Expression	CI at ED50	Description
CNE2*	High	0.36	Synergism
MCF7*	High	0.87	Synergism
YCC16*	High	0.79	Synergism
SNU1	High	1.65	Antagonism
CNE1	Low	1.26	Antagonism
HONE1	Low	0.91	Additive
HK1	Low	1.38	Antagonism
SKBR3	Low	1.57	Antagonism
T47D	Low	1.28	Antagonism
AGS	Low	1.18	Antagonism

Combination treatment of masitinib and gemcitabine was tested on nasopharyngeal (n = 4), breast (n = 3) and gastric (n = 3) cancer cell lines. Combination index (CI) were generated by Calcusyn software for combination of gossypol and gemcitabine. CI <1 is indicated as synergistic of the combination treatment.

* indicates gemcitabine resistance cell lines. High/low Bcl-2 expression is defined by the presence or absence of Bcl-2 expression based on immunoblot of the gene.

### Reversal of Gemcitabine Resistance by Gossypol was Associated with Up-regulation of Noxa and Down-regulation of Bcl-2 and Bcl-xl

**Figure 5:**
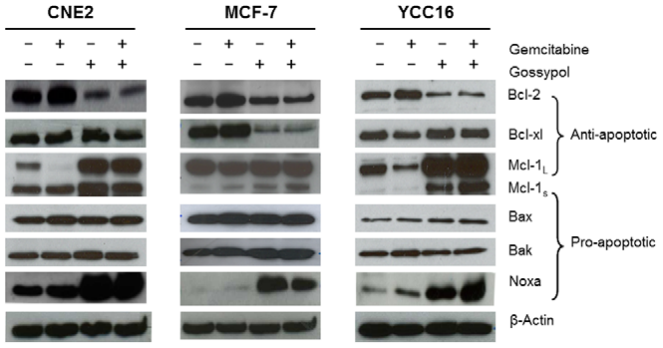
Mechanism of reversal of gemcitabine resistance by gossypol in high Bcl-2 cell lines. Immunoblots of apoptotic protein expressions in gemcitabine resistant cell lines (GEM-R) when treated with combination of gossypol and gemcitabine for 48 hours. The results shown are representative of two independent experiments.

To investigate the underlying molecular mechanisms of synergistic interaction between gossypol and gemcitabine, we examined the changes of pro-apoptotic and anti-apoptotic proteins in high Bcl-2 expression GEM-R cell lines (YCC16, CNE2 and MCF7). As shown in Figure 5, anti-apoptotic proteins (Bcl-2 and/or Bcl-xl) were up-regulated in all Bcl-2 overexpressing GEM-R cell lines after gemcitabine treatment. In contrast, treatment with gossypol led to the down-regulation of anti-apoptotic proteins (Bcl-2 and/or Bcl-xl) and up-regulation of pro-apoptotic protein, (Noxa and Mcl-1_s_) (Figure 5). An overall decrease in Bcl-2 expression was also observed in all GEM-R cell lines treated with gossypol or combined drug treatment. However, down-regulation of Bcl-xl was also observed in MCF-7 when cells were treated with either gossypol or combined drug treatment. An increase in the cleavage of pro-apoptotic Mcl-1_S_ expression in all Bcl-2 overexpressing GEM-R cell lines was observed after combined drug treatment. However expressions of Bax and Bak remained unchanged after drug treatment. Collectively, these observations suggested that gossypol reversal of gemcitabine resistance involves the down-regulation of anti-apoptotic proteins (Bcl-2 and/or Bcl-xl) and up-regulation of pro-apoptotic proteins (Noxa and Mcl-1_S_).

### Significant Changes in Apoptotic Pathway Contributed to Differential Response to Gemcitabine and Gossypol

**Figure 6:**
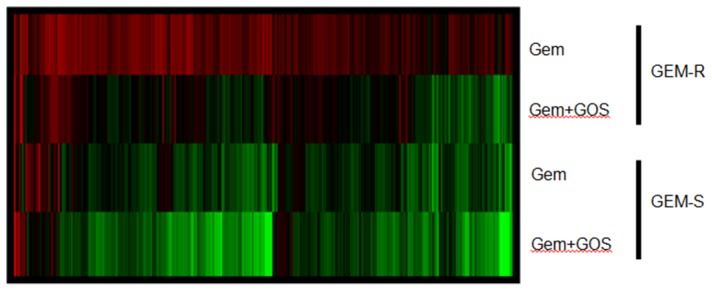
Gene expression analysis in response to gemcitabine and combined treatment. Heatmap of 2702 significant differentially expressed genes in gemcitabine resistant, GEM-R and gemcitabine sensitive, GEM-S treated with gemcitabine alone (Gem) or in combination with gossypol (Gem+GOS) relative to untreated matched control cell lines. The expression levels of each gene are higher and lower than control is depicted in red and green, respectively.

Whole genome expression profiling was carried out in SNU1 (GEM-S) and CNE2 (GEM-R) to evaluate alternative mechanisms for their differences in drug response to gemcitabine alone and gemcitabine/gossypol combination (Figure 6). A total of 2702 genes were differentially expressed in pre- and post-treatment samples and was mapped to 61 enriched biological processes using KEGG pathway analysis. The most significant biological processes regulated by these genes include spliceosome, cell cycle, pyrimidine metabolism, p53 signalling and apoptotic pathways (see Supporting Information; Table S1). Treatment with gemcitabine predominantly led to up-regulation of these treatment response genes in CNE2 (GEM-R) but a down-regulation in SNU1 (GEM-S) (Figure 6), suggesting that the up-regulation of these genes involving in p53 and apoptotic pathways may contribute towards gemcitabine resistance. In addition, nearly half of these treatment response genes were down-regulated in CNE2 (GEM-R) after treatment with gossypol and gemcitabine with minimal changes observed in SNU1 (GEM-S).

### Decreased Anti- and Pro-apoptotic Gene Expressions in GEM-R and GEM-S Cell Lines when Gossypol was Combined with Gemcitabine Treatment

To further evaluate genes that may be responsible for gemcitabine sensitivity and synergism to combined treatment with gemcitabine and gossypol, we decided to focus only on apoptosis related genes in the KEGG pathway that were differentially modulated after treatment of gemcitabine alone and after combined therapy. We identified 65 genes (31 anti-apoptosis and 34 pro-apoptosis) in the apoptotic pathway that were significantly regulated in CNE2 (GEM-R), and 49 genes (23 anti-apoptosis and 26 pro-apoptosis) that were significantly regulated in SNU1 (GEM-S) (see Supporting Information; Table S2).

**Figure 7:**
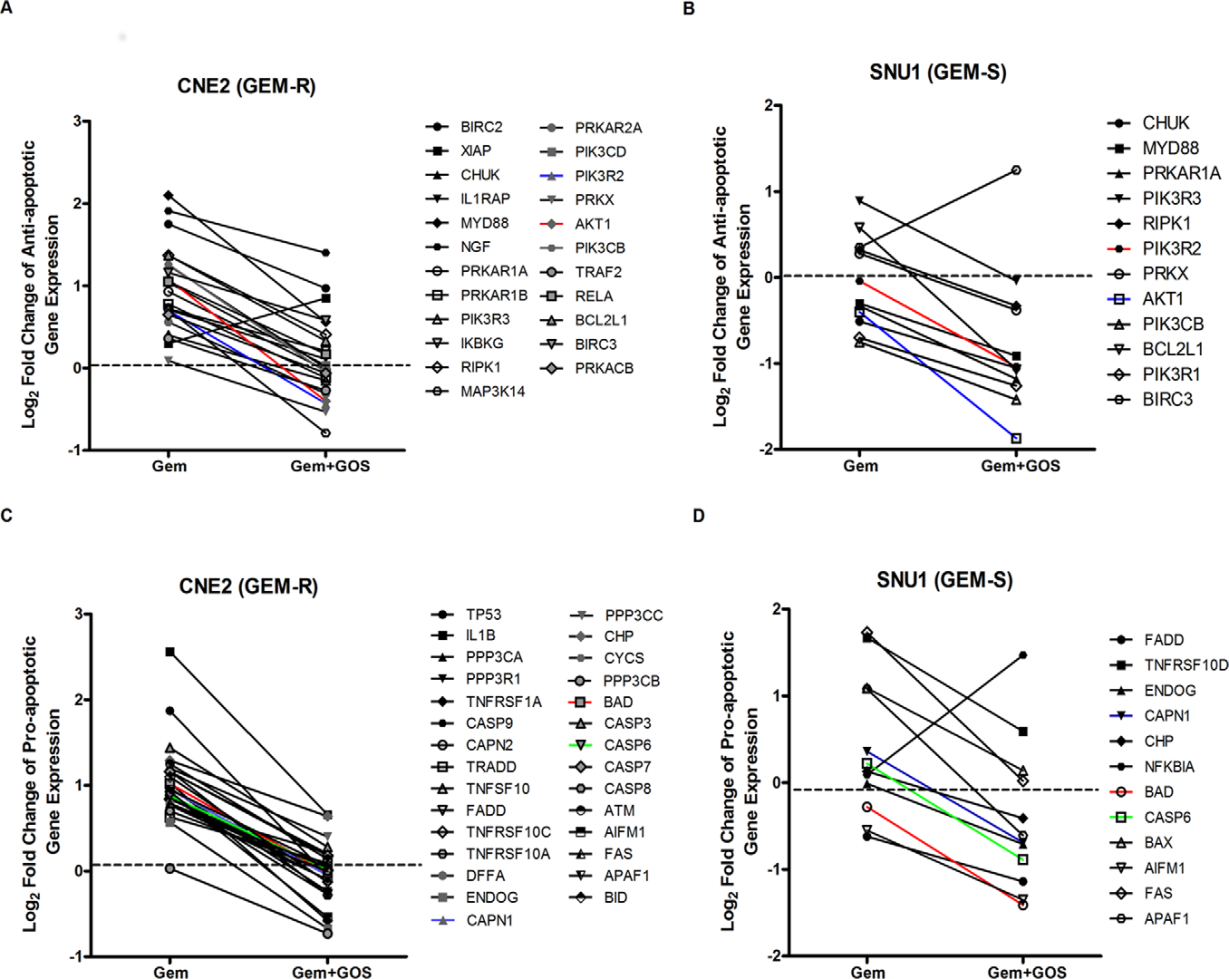
Anti- and pro-apoptotic gene expressions in GEM-R and GEM-S cell lines after combined treatment. The relative log_2_ fold change for anti-apoptotic gene cluster (A–B) and pro-apoptotic gene cluster (C–D), treated with gemcitabine alone (Gem) and in combination with gossypol (Gem+GOS). Only genes that were significantly differentially expressed from control untreated group are represented in the graph. All values on the y-axis were compared to the untreated controls of respective cell lines. Values above zero indicated genes that were up-regulated in its expression. Values below zero indicated genes that were down-regulated in its expression and values at zero indicated no change to gene expression. The log_2_ levels of associated genes were normalized to the untreated sample.

In CNE2 (GEM-R), we found that most of the anti-apoptotic genes were significantly up-regulated when cells were treated with gemcitabine (Figure 7A). Conversely, most of these anti-apoptotic genes were down-regulated when SNU1 (GEM-S) was treated with gemcitabine alone (Figure 7B). It was also observed that the gene expressions of the majority of these anti-apoptotic genes were significantly decreased when gossypol was added to gemcitabine in CNE2 (GEM-R) (Figure 7A). Similarly anti-apoptotic genes that were downregulated in SNU1 (GEM-S) after gemcitabine treatment, were also found to be futher downregulated when cells were treated with combination therapy (Figure 7B). However, there were also more anti-apoptoptic genes, initially up-regulated by gemcitabine but were downregulated in CNE2 (GEM-R) (11 genes) after combination of gossypol and gemcitabine, compared to SNU1 (4 genes) (Figure 7A and B).

Similar trend of decreased pro-apoptotic genes expressions was observed in both cell lines treated with combination drug therapy compared to cell lines treated with gemcitabine alone (Figure 7C and D). However, despite the decreased in pro-apoptotic gene expression, there were more pro-apoptotic genes in CNE2 (GEM-R) (15/29 genes) that remained up-regulated after combined treatment (Figure 7C), compared to SNU1 (GEM-S) (4/12 genes) in the same treatment (Figure 7D). It should be noted that only the gene expression of NFKBIA was found to be up-reglated in SNU1 (GEM-S) after combination therapy when compared to its gene expression during gemcitabine treatment (Figure 7D).

### Validation of Anti-apoptotic Gene Cluster in GEM-R and Pro-apoptotic Gene Cluster in GEM-S

**Figure 8:**
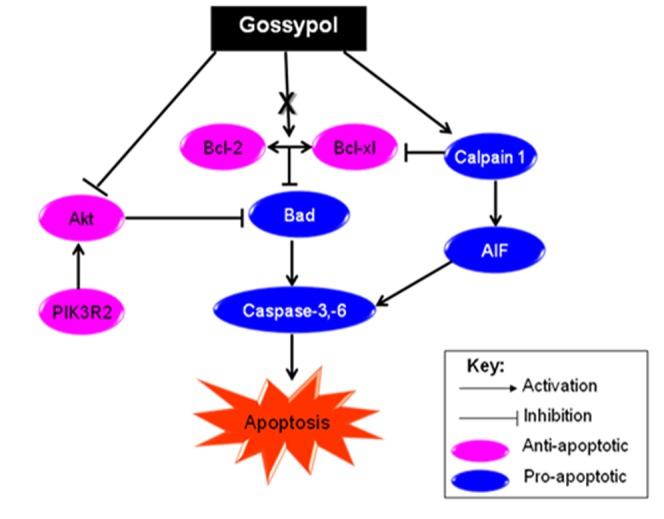
The alternative apoptosis pathway for gossypol inducing apoptosis.

**Figure 9:**
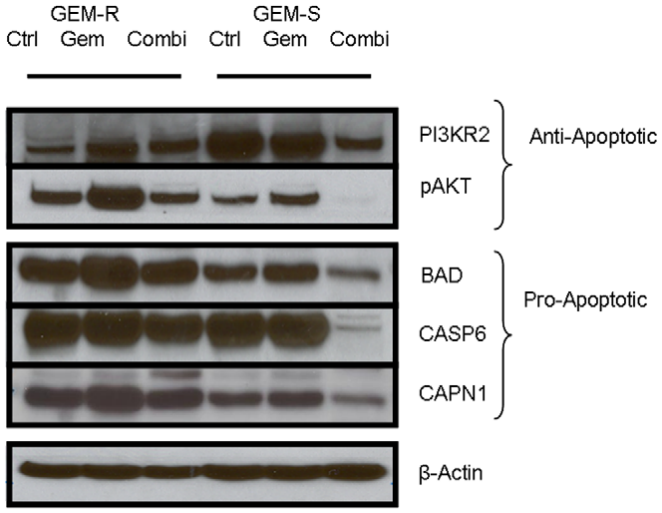
Validation of anti-apoptotic gene cluster in GEM-R and pro-apoptotic gene cluster in GEM-S. Immunoblot of anti-apoptotic (pAKT and PI3KR2) and pro-apoptotic (BAD, CASP6 and CAPN1) genes in GEM-R cell line and GEM-S cell lines. Cells were treated with gemcitabine and combination with gossypol for 48 hours. The results shown are representative of two independent experiments.

To validate the cluster of anti- and pro-apoptotic genes in CNE2 (GEM-R) and SNU1 (GEM-S) cell lines shown in Figure 7, we investigated 5 selected genes in the KEGG apoptosis pathway that may have important role in apoptosis in response to gemcitabine and gossypol treatment (Figure 8). Similar to gene expression assay, anti-apoptotic PIK3R2 and pAKT protein expression were found to be up-regulated when CNE2 (GEM-R) cell line was treated with gemcitabine, with a reduction in protein level observed only in pAKT when cells were treated with combination treatment (Figure 9). Gemcitabine treated SNU1 (GEM-S) cell line showed minimal changes in PIK3R2 and pAKT protein expression when compared to untreated cells, but combination treatment with SNU1 (GEM-S) cell line resulted in significant reduction of PIK3R2 and pAKT protein expression (Figure 9).

We observed that there was a minor increase in the protein expression of pro-apoptotic genes, BAD, CASP6 and CAPN1 after gemcitabine treatment in both CNE2 (GEM-R) and SNU1 (GEM-S) cell line (Figure 9). BAD, CASP6 and CAPN1 were found to be downregulated during combination treatment in GEM-S cell line but not GEM-R cell line (Figure 9). These observations concurs with the findings in Figure 5, and suggest that alternative Bcl-2 pathways that involved AKT, PI3K, BAD, CASP6 and CAPN1 may help explain differential response to gemcitabine and synergistic interactions in combination treatment of gemcitabine and gossypol.

## Discussion

High Bcl-2 expression has been correlated with poor clinical prognosis in cancer patients [29] as well as resistance to conventional chemotherapeutic drugs [30]. Han et al reported that the overexpression of Bcl-2 has been shown to be significantly associated with decreased sensitivity to gemcitabine treatment, and that its expression could be utilised as a predictor factor for the efficacy of gemcitabine treatment in cancer patients [12]. In vitro studies has also revealed a direct correlation with high Bcl-2 cellular content and resistance to gemcitabine in pancreatic carcinoma cell lines [11]. However, there was conflicting report observing no correlation between Bcl-2 and gemcitabine sensitivity in pancreas, prostate, lung and breast cancer [31].

In our study, we found that nasopharyngeal, breast and gastric cancer cell lines resistant to gemcitabine had higher Bcl-2 expression, and treatment with gemcitabine resulted in an up-regulation of anti-apoptotic Bcl-2 or Bcl-xl. These observations suggested that the level of gene expression of anti-apoptotic Bcl-2 and Bcl-xl was important in gemcitabine resistance. In support of this finding, Schniewind et al found a significant inverse correlation between Bcl-xl expression and gemcitabine induced apoptosis [32], and that increased Bcl-xl expression inhibits cellular apoptosis to chemotherapeutic drugs [33].

Several reports had demonstrated the effectiveness of gossypol in targeting cancer cells with high level of Bcl-2 and its family members, by increasing cellular apoptosis when used as a single agent [19] or in combination with gemcitabine [34]. Macoska et al showed synergistic interaction in bladder cancer cell lines treated with gossypol in combination with gemcitabine or carboplatin, resulting in an increase in apoptosis via the decreased expression of pro-survival Bcl-xl and Mcl-1 and increased expression of pro-apoptotic genes [34]. However, one ought to be mindful that not all Bcl-2 overexpressing cell lines may benefit from combining gossypol with gemcitabine. In our study, potential antagonistic effect of gossylpol and gemcitabine was observed in SNU1, a cell line that had high level of Bcl-2 but was sensitive to gemcitabine, suggesting an alternative Bcl-2 pathway in gemcitabine response [35], [36].

We observed that GEM-R cell lines with high Bcl-2 had up-regulated pro-apoptotic Noxa and down-regulated Bcl-2 or Bcl-xl expression when treated with either gossypol or in combination with gemcitabine (Figure 5). It has been suggested that gossypol targets Bcl-xl by inhibiting in anti-apoptotic activity, which directly result in the up-regulation of pro-apoptotic Noxa and Puma expression [37]. The activation of Noxa could also lead to BH3 motif-dependent localization to mitochondria and interaction with anti-apoptotic Bcl-2 family members, resulting in the activation of apoptosis [38]. Moreover, studies have demonstrated that up-regulated pro-apoptotic genes as well decreased expression of anti-apoptotic genes were able to increase gossypol induced apoptosis in several types of cancer cells [39], [40]. We also showed that Mcl-1_S_ was increased in a GEM-R cell line treated with gossypol or combined treatment (Figure 5). This observation can be supported by Meng et al. where gossypol was also found to elevate Mcl-1 protein levels [37], suggesting that Mcl-1_S_ could be regulated either at alternative splicing during post transcriptional level or caspase-cleaved during post-translational level, both of which have pro-apoptotic function [41].

The differences in the regulation of gene expression during single and dual drug therapy in GEM-R cell lines with high Bcl-2 could be attributed to the cell type specific effects of gossypol on Bcl-2, suggesting that there may be alternatives to the Bcl-2 independent pathways in the response to drug treatment. Results in this study remained consistent with other reports where gossypol exerts its antitumour activity through functional inhibition rather than affecting protein expression levels of Bcl-2 and Bcl-xl [42], [43]. Oliver et al have also proposed that there might be two potential models of gossypol action, where gossypol may act directly by binding to Bcl-2 or Bcl-xl, or interact indirectly with pro-apoptotic Bcl-2 family members [21]. Interestingly, similar synergistic drug interaction was not observed in gemcitabine sensitive cell lines, possibly due to the cytotoxicity of gemcitabine in these cell lines [44].

Whole-genome expression profiling in 2 highly expressing Bcl-2 cell lines revealed that gemcitabine treated cell lines had different gene expression profile relative to gemcitabine response. It was found that GEM-R cell line appeared to have more up-regulated genes compared to a cell line that was sensitive to gemcitabine. This observation appeared to be consistent with findings of more anti-apoptotic genes were up-regulated after gemcitabine treatment in a GEM-R cell line compared to one that was sensitive to the drug (Figure 7). However, when both cell lines were treated with drug combination, there appeared to be a general down-regulation of gene expressions in both high Bcl-2 expressing cell lines, regardless of their gemcitabine sensitivity. Interestingly, despite the downward trend of genes expressions observed in both cell lines during combination treatment, it should be noted that the decreased in gene expression in GEM-R cell line, reached similar levels to those in the untreated control cells. In contrast, there was a significant down-regulation of gene expression in both anti- and pro-apoptotic genes compared to untreated controls in gemcitabine-sensitive cell line treated with dual drug therapy. A possible explanation of the synergistic difference observed in these cell lines may be explained in the less than drastic drop of gene expressions of pro-apoptotic genes observed in gemcitabine-resistant cell line. As the expressions of pro-apoptotic genes remained comparable to untreated controls, these cells may still have the ability to induce apoptosis. In contrast, the expressions of pro-apoptotic genes were more down-regulated than their untreated controls in the gemcitabine-sensitive cell line, possibly contributing to its loss of function in apoptotic processing involved in synergism. Furthermore, the low level of both pro- and anti-apoptotic genes expression observed in gemcitabine-sensitive cell line treated with either gemcitabine or when combined with gossypol, could also account for the lack of synergism during dual drug therapy or may suggest an alternative independent Bcl-2 pathway in gemcitabine sensitivity [45].

Our study demonstrated that despite the down-regulation of anti-apoptotic genes in gemcitabine-sensitive cell line after combination therapy, the decreased expression of pro-apoptotic genes appeared to be the major driver for the antagonism observed during combination drug therapy. A possible explanation of this observation may lie in the importance of pro-apoptotic genes regulation and their interaction with Bcl-2 related genes in promoting synergism. Youle et al underlie the important role of pro-apoptotic family members in regulating caspase activation, and that the anti-apoptotic family members, such as BCL-2 and BCL-XL can act to inhibit these pro-apoptotic genes function in inducing apoptosis [46]. Gene expression analysis also revealed that the expression of pro-apoptotic genes such as Bax, Bak, AIF, NOXA1, CAD, PARP and cytochrome c were increased when cancer cells were treated with gossypol [47]. Taken together, these results suggested that the mode of action of gossypol in combination therapy depends mainly on pro-apoptotic genes to drive the synergistic interaction in combination therapy with gemcitabine.

To validate our findings in gene expression, we selected 5 genes to investigate their protein expression in response to gemcitabine and in combination with gossypol. We observed an increase in anti-apoptotic PIK3R2 and pAKT gene expression in gemcitabine treated GEM-R cell line but decrease in their expression when cells were treated in combination therapy (Figure 7, 9). This reduction of pAKT by gossypol during combination therapy remained consistent with previous reports [48]. Similar observations changes in gene expression were noted at both the RNA and protein level, where pro-apoptotic Bad, CAPN1 and CASP6 expression were lowered after combination treatment in GEM-S cell line but remained unchanged in GEM-R cell line (Figure 9). The interactions of these 5 genes and their roles in the apoptosis have been illustrated in Figure 5. Despite having high Bcl-2, gemcitabine-sensitive cell line (GEM-S) showed no change in gene expression after gemcitabine treatment in all 5 genes, suggesting that there may be an alternative Bcl-2 pathway in gemcitabine sensitivity (Figure 9).

The role of BAD as a pro-apoptotic gene is well established [46]. However, there have been very few reports on caspase 6 and calapin 1 (CAPN1), especially in the context of their involvement in combination therapy. As caspase-6 is required for the activation of caspases-3, -7, and -9 [49], the loss of its gene expression could significantly inhibit the induction of apoptosis. Similarly, CAPN1, a ubiquitous cysteine protease, has been shown to cleave Bax and BID resulting in the release of cytochrome c that promotes apoptosis [50]. Calpain cleavage has also been shown to activate caspase-7, caspase-10 and caspase-12 leading to apoptosis [51], [52]. Antagonistic interaction was observed in GEM-S cell line treated with combination therapy despite the loss of anti-apoptotic p-Akt. It is likely that the down-regulation of 3 important pro-apoptotic genes (Bad, caspase 6 and CAPN1) may play a more significant role in determining synergism in combination treatment between gemcitabine and gossypol.

### Conclusion

In conclusion, we found that gemcitabine resistance is associated with high Bcl-2 protein expression. Gossypol, a Bcl-2 family inhibitor, is able to overcome resistance to gemciatbine through synergistic interaction of both drugs involving the Bcl-2 family of genes via the up-regulation of pro-apoptotic genes, Noxa and down-regulation of anti-apoptotic genes (Bcl-2 and Bcl-xl). In contrast, cell lines that was sensitive to gemcitabine regardless of their Bcl-2 levels, showed antagonism to combination treatment with gossypol. Nonetheless, our findings suggested clinical application for treatment management in patients bearing gemcitabine resistant tumours that expressed Bcl-2, and may benefit from the synergistic interaction of combination therapy with gossypol.

## Supporting Information

Figure S1 Isobolograms showing the interactions bwtween gemcitabine and gossypol. These results were generated by CalcuSyn software for (A) CNE2, (B) MCF7, (C) YCC16, (D) SNU1, (E) CNE1, (F) HONE1, (G) HK1, (H) SKBR3, (I) T47D and (J) AGS. The ED points located on the lower left of the diagonal indicating synergism.
(TIF)

Table S1 2702 significant genes corresponding to 61 significant pathways differentially expressed between the treated and untreated cell lines.
(XLS)

Table S2 Pro- and anti-apoptosis genes that are significantly regulated in treated and untreated cells lines.
(XLS)
