# Supplementary figures and images for: Combination Therapy with Gossypol Reveals Synergism against Gemcitabine Resistance in Cancer Cells with High BCL-2 Expression
Source: PLoS One. 2012 Dec 4;7(12):e50786. doi: 10.1371/journal.pone.0050786 (PMC3514173; doi:10.1371/journal.pone.0050786)

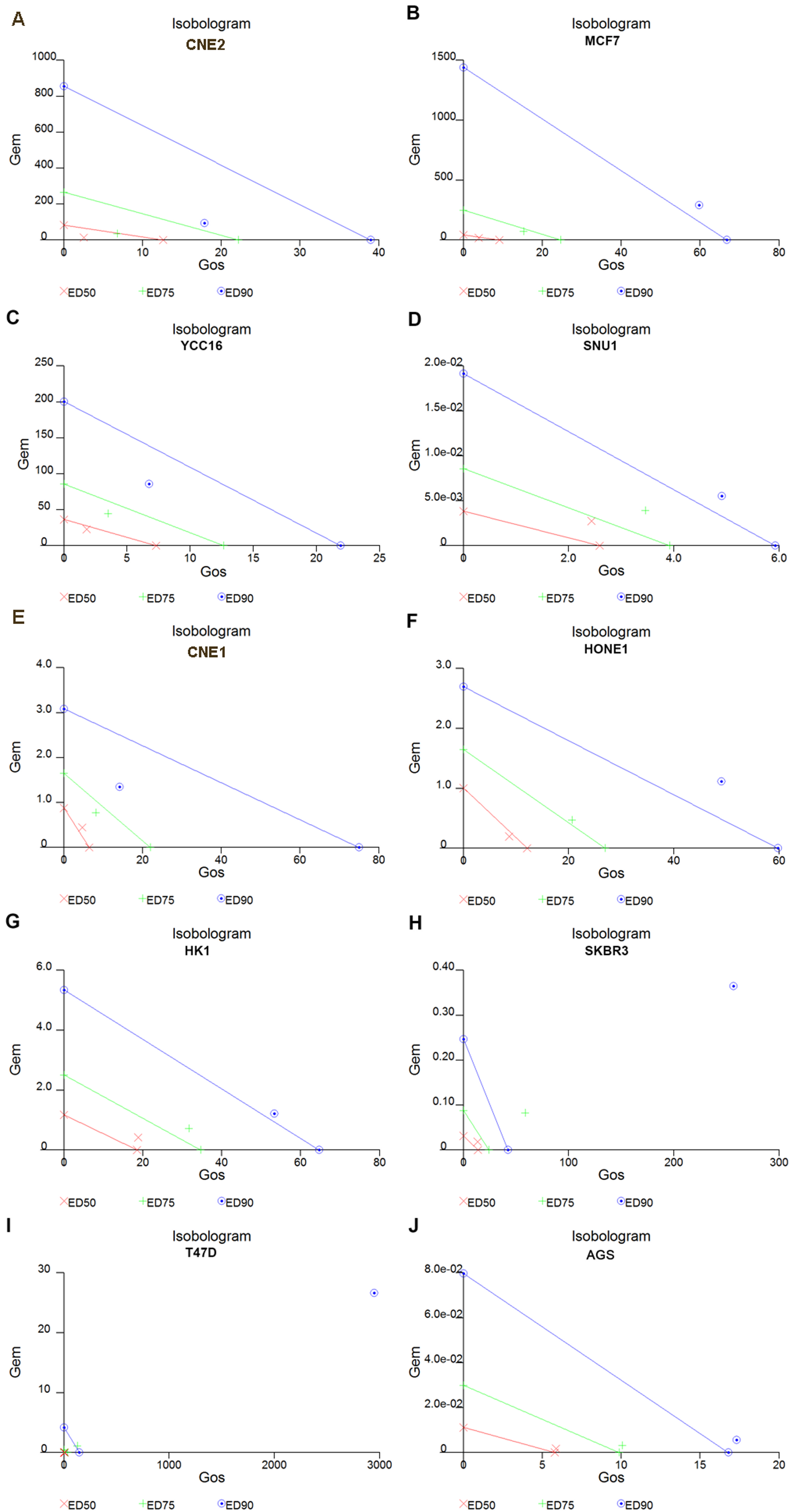

Supplement: Figure S1 — Isobolograms showing the interactions bwtween gemcitabine and gossypol. These results were generated by CalcuSyn software for (A) CNE2, (B) MCF7, (C) YCC16, (D) SNU1, (E) CNE1, (F) HONE1, (G) HK1, (H) SKBR3, (I) T47D and (J) AGS. The ED points located on the lower left of the diagonal indicating synergism. (TIF) [file pone.0050786.s001.tif]
